# Urinary Hepcidin Levels in Iron-Deficient and Iron-Supplemented Piglets Correlate with Hepcidin Hepatic mRNA and Serum Levels and with Body Iron Status

**DOI:** 10.1371/journal.pone.0136695

**Published:** 2015-08-31

**Authors:** Robert Staroń, Rachel P. L. Van Swelm, Paweł Lipiński, Anna Gajowiak, Małgorzata Lenartowicz, Aleksandra Bednarz, Małgorzata Gajewska, Marek Pieszka, Coby M. M. Laarakkers, Dorine W. Swinkels, Rafał R. Starzyński

**Affiliations:** 1 Institute of Genetics and Animal Breeding PAS, Department of Molecular Biology, Jastrzębiec, Poland; 2 Department of Genetics and Evolution, Institute of Zoology, Jagiellonian University, Kraków, Poland; 3 Warsaw University of Life Sciences, Faculty of Veterinary Medicine, Department of Physiological Sciences, Warsaw, Poland; 4 Department of Animal Nutrition & Feed Science, National Research Institute of Animal Production, Kraków, Poland; 5 Department of Laboratory Medicine (LGEM 830), Radboud University Medical Centre, Nijmegen, The Netherlands; Lady Davis Institute for Medical Research/McGill University, CANADA

## Abstract

Among livestock, domestic pig (*Sus scrofa*) is a species, in which iron metabolism has been most intensively examined during last decade. The obvious reason for studying the regulation of iron homeostasis especially in young pigs is neonatal iron deficiency anemia commonly occurring in these animals. Moreover, supplementation of essentially all commercially reared piglets with iron entails a need for monitoring the efficacy of this routine practice followed in the swine industry for several decades. Since the discovery of hepcidin many studies confirmed its role as key regulator of iron metabolism and pointed out the assessment of its concentrations in biological fluids as diagnostic tool for iron-related disorder. Here we demonstrate that urine hepcidin-25 levels measured by a combination of weak cation exchange chromatography and time-of-flight mass spectrometry (WCX-TOF MS) are highly correlated with mRNA hepcidin expression in the liver and plasma hepcidin-25 concentrations in anemic and iron-supplemented 28-day old piglets. We also found a high correlation between urine hepcidin level and hepatic non-heme iron content. Our results show that similarly to previously described transgenic mouse models of iron disorders, young pigs constitute a convenient animal model to explore accuracy and relationship between indicators for assessing systemic iron status.

## Introduction

Hepcidin is a 25-amino acid peptide hormone that regulates iron balance. It is synthesized mainly by hepatocytes, released into the circulation, and excreted in urine by the kidneys [1]. Increased levels of iron in the plasma and in iron storage sites stimulate the production of hepcidin [2], whereas iron deficiency down-regulates its synthesis [3]. Other studies have revealed that hepcidin inhibits cellular efflux of iron by binding to ferroportin, the sole cellular iron exporter, which is present on the surface of macrophages of the reticuloendothelial system, duodenal enterocytes and hepatocytes. The binding precedes subsequent internalization and degradation of ferroportin in lysosomes [1]. The above-described feedback regulation between iron and hepcidin controls the appropriate physiological concentration of iron in the plasma and its bioavailability for many vital processes including erythropoiesis. Accordingly, as previously shown, hepcidin deficiency leads to iron overload [4] and the overexpression of hepcidin causes severe iron deficiency in transgenic mice [5]. After being identified as a key iron regulatory peptide, hepcidin has been put forward as a potentially valuable diagnostic marker for iron-related disorders [6]. In this regard, a number of analytical methods have been developed in order to assess hepcidin concentration in serum and in urine [7], [8].

Iron deficiency anemia (IDA) is a worldwide human pathology occurring also, to a greater or lesser extent, in newborn pigs of all breeds [9], [10]. Considering that the red blood cell pool is the largest functional iron compartment in the body, it is comprehensible that insufficient iron stores and inadequate dietary intake observed in piglets, result for those animals in disturbed iron balance and reduced, iron supply for their erythropoiesis. Therefore, to counteract the development of anemia in young pigs, an exogenous iron source must be administered routinely, with the most common method being an intramuscular injection of a large amount of iron dextran (150 mg Fe/kg piglet) [11]. This treatment dramatically shifts iron status of piglets from severe iron depletion to iron repletion, or even a slight iron overload [12], [13], [14], [15]. Therefore, anemic and iron-supplemented piglets are considered to be a suitable animal models of the opposite profiles of iron metabolism and can be successfully employed to explore the usefulness and accuracy of various indicators (such as hepcidin level in urine or in plasma, biochemical iron parameters in plasma and RBC indices) in the estimation of systemic iron status.

In this study, we validated for the first time the usefulness of a combination of weak cation exchange chromatography and the time-of-flight mass spectrometry (WCX-TOF MS) methods for a reliable quantification pig hepcidin-25 in urine by measuring this parameter in 28-day old piglets. The animals were divided and appropriately supplemented (or not) to have two groups of animals with an opposite iron status: iron-deficient (anemic) and iron-overloaded (iron-supplemented) piglets. In both groups of animals we demonstrate that urine hepcidin-25 concentrations strongly correlate with hepatic hepcidin mRNA abundance, plasma hepcidin-25 levels, iron transferrin saturation and non-heme liver iron levels. Our study shows how comprehensive evaluation of hepcidin expression may help in the diagnosis of iron deficiency and its treatment in piglets.

## Materials and Methods

### Ethics Statement

Use of animals in the experiment and all procedures were approved by the Third Local Ethical Committee on Animal Testing at the Warsaw University of Life Sciences—SGGW in Warsaw (permission no. 55/2012).

### Animals, experimental design, and samples collection

Experiments were conducted at the pig farm Brzezie belonging to the National Research Institute of Animal Production (Balice, Poland). A total of 14 Polish Landrace x Polish Large White piglets housed in standard conditions (approx. 70% humidity and a temperature of 22°C in standard cages with straw bedding) were used in the experiments. During the 28-day experiment sows were allowed to nurse their piglets. The feed (Prestarter, was manufactured at the feed mill of the Experimental Station of the National Research Institute of Animal Production in Brzezie; iron content 0.75 mg Fe/kg feed mixture) was offered to piglets from day 3 to day 28. Piglets were taken from 3 litters delivered by 3 multipara sows. They were allotted to two experimental groups (7 piglets per group) on the basis of balanced body weight (b.w.) at birth: A) control piglets with no iron supplementation B) piglets routinely supplemented by intramuscular injection with 150 and 40 mg Fe/kg b.w. on days 3 and 21 postpartum, respectively. Iron was administered to piglets by intramuscular injection in the neck in the form of iron dextran (FeDex), a complex of ferric ions with low molecular weight dextran (Ferran 100, Vet-Agro, Lublin, Poland). Blood and urine as well as liver samples were taken for analyses from all piglets on day 28 of life. Blood was drawn by venipuncture of the jugular vein (*Vena jugularis externa*) into tubes coated with heparin as an anticoagulant. Blood was immediately spun down (4°C, 2000 rpm, 10 min) to collect plasma. Piglets were euthanized by the intracardiac injection of Morbital (Biowet, Puławy, Poland). Urine was collected *post mortem* by urinary bladder puncture. Plasma and urine samples were immediately aliquoted and stored at -80°C. Liver samples were frozen in liquid nitrogen and kept at -80°C until they were analyzed for hepcidin mRNA expression and non-heme iron content.

### Real-time quantitative reverse transcription-polymerase chain reaction analysis of hepatic hepcidin mRNA abundance

Total cellular RNA was extracted from liver samples (20 mg) using Trizol reagent (Invitrogen) according to the manufacturer’s protocol. Two micrograms of total DNAse-treated RNA were used for reverse transcription using a Transcriptor First Strand cDNA Synthesis Kit (ROCHE, Switzerland). Real-time quantitative RNA analysis was performed in a Light Cycler U96 (Roche Diagnostics, Mannheim, Germany) using the respective pairs of oligonucleotide primers: *hepc* F: 3’ aagacagctcacagacctcc 5’; *hepc*R: 5’ ctacgtcttgcagcacatcc 3’, and *s18*F: 5’ aggaaagcagacatcgacct 3’, *s18*R: 5’ acctggctgtacttcccatc 3’. Amplified products were detected using SYBR Green I (Roche Diagnostics) as described previously [16]. To confirm amplification specificity, the PCR product was subjected to melting curve analysis and agarose gel electrophoresis. Light Cycler U96 Software was used for data analysis. Expression was normalized relative to that of control transcript encoding *18S* rRNA selected among other reference genes using NormFinder software (http://www.mdl.dk/publicationsnormfinder.htm).

### Hemoglobin level and quantitative hepatic non-hem iron measurement

Hemoglobin level (HGB), was determined using an automated ADVIA 2010 analyzer (Siemens, Germany). The non-heme iron content of liver fragments (100 mg) was determined by acid digestion of the samples at 100°C for 10 h, followed by colorimetric measurement of the absorbance of the iron-ferrozine complex at 560 nm as described previously (Torrance and Bothwell, 1980).

### Hepatic iron staining

Non-heme iron deposits were analysed using Accustain Iron Deposition Kit (Sigma Aldrich). Briefly, liver samples were excised immediately after sacrifice and fixed in Bouin’s solution for 24h, then stored in 70% ethanol. After embedding in paraffin, the samples were cut into 7-μm sections with a microtome (Reichert-Jung, Germany). The sections were placed on a slide, deparaffinised and hydrated tissues were incubated with working solution containing Perls’ Prussian Blue for 30 min. Slide were counterstained with pararoseaniline solution for 2 min and analysed under standard light microscopy (Olympus, type CH2).

### Plasma iron concentration and transferrin saturation

Plasma iron concentration and total iron binding capacity (TIBC) were determined by colorimetric measurement of the absorbance of the iron-chromazurol complex at 630 nm according to manufacturer protocol (Alpha Diagnostic, Poland). Percent of transferrin saturation (TSAT) was then calculated according to the following formula: TSAT = (plasma iron/TIBC) × 100.

### Plasma hepcidin-25 quantification

Piglet plasma hepcidin-25 measurements were performed by a combination of weak cation exchange chromatography and time-of-flight mass spectrometry (WCX-TOF MS), as described previously for human and porcine plasma samples [17], [7], [15]. In short, acetonitril and a synthetic human hepcidin-25 standard (Peptide International Inc.) were added to the samples. The resulting solution was mixed and centrifuged at 27500 x g for 5 min., after which the supernatant was collected. Weak cationic exchange beads (Macro-Prep Support Beads; Bio-Rad Laboratories) and binding buffer were added to the supernatant, which was mixed thoroughly. The solution was incubated for 15 min. on the rollerbank at RT to allow hepcidin to bind to the beads. Next, the beads were washed three times and the bound hepcidin was eluted from the beads using elution buffer consisting of acetonitril and trifluoroacetic acid. The eluted fraction was spotted onto a MSP 96 polished steel target plate (Bruker Daltonics) followed by an energy-absorbing matrix (5 mg/mL α-cyano-4-hydroxy cinnamic acid (Bruker Daltonics). Peptide spectra were generated on a Microflex LT matrix-enhanced laser desorption/ionisation TOF MS platform (Bruker Daltonics). The concentration of pig hepcidin-25 was calculated by comparing its mass peak height with that of the internal standard. Piglet plasma hepcidin-25 concentrations were expressed as nmol/L (nM). The lower limit of detection of this method was 1 nM.

### Urine Hepcidin-25 Quantification

For the first time piglet urine hepcidin-25 measurements were performed by a combination of weak cation exchange chromatography and time-of-flight mass spectrometry (WCX-TOF MS), as described previously for pig plasma [15].

To demonstrate that the observed mass peak in urine was indeed hepcidin-25, we incubated a urine sample with anti-hepcidin O/N at 4°C, before measuring using WCX-TOF MS [18].

### Statistical Analysis

Data are presented as mean values ± SD. Statistical analysis of results was performed by Student T-test. using Statgraphics 5.1 program (Manugistics, USA). Statistically significant differences between parameters of piglets from control group *vs* iron supplemented group on 28^th^ day of experiment were denoted by one and two asterisks at P≤0.05 and P≤0.01, respectively. Correlation between quantitative variables were assessed using Spearman rank correlation test.

## Results and Discussion

Anemia is one of the World Health Organization's top 10 target diseases for treatment and prevention [19] and dietary iron deficiency is one of the most frequently occurring causes of this disorder. In consequence, iron deficiency anemia (IDA) has been recognized as a widespread nutritional deficiency in humans with high prevalence in neonatal period [20]. Our previous studies have indicated that young piglets provide a convenient model for exploring molecular mechanisms underlying neonatal IDA as well as for testing the effectiveness of various iron supplementation strategies [14], [15]. Thus, the expression of hepcidin, one of the most important factors in the pathogenesis of disorders of iron metabolism was investigated in both anemic and iron supplemented piglets [14], [15]. We showed that it was induced at the mRNA level in the liver of piglets supplemented intramuscularly with a large amount of iron dextran, a procedure widely used in the swine industry [14]. We have also found substantially elevated levels of hepcidin-25 in the plasma of those piglets using a combination of weak cation exchange chromatography and time of-flight mass spectrometry (WCX-TOF MS) [15]. In contrast, in anemic piglets hepcidin-25 was barely detectable in the plasma [15]. From these studies we concluded that hepcidin is a promising marker for the diagnosis and management of IDA in newborn piglets. Similar conclusions about the usefulness of hepcidin assessment in the serum have been drawn from studies on mice [21] and humans [7].

Hepcidin was originally purified from human urine [22] and before being identified as the key iron regulatory peptide, it was perceived as an antimicrobial factor.

**Fig 1:**
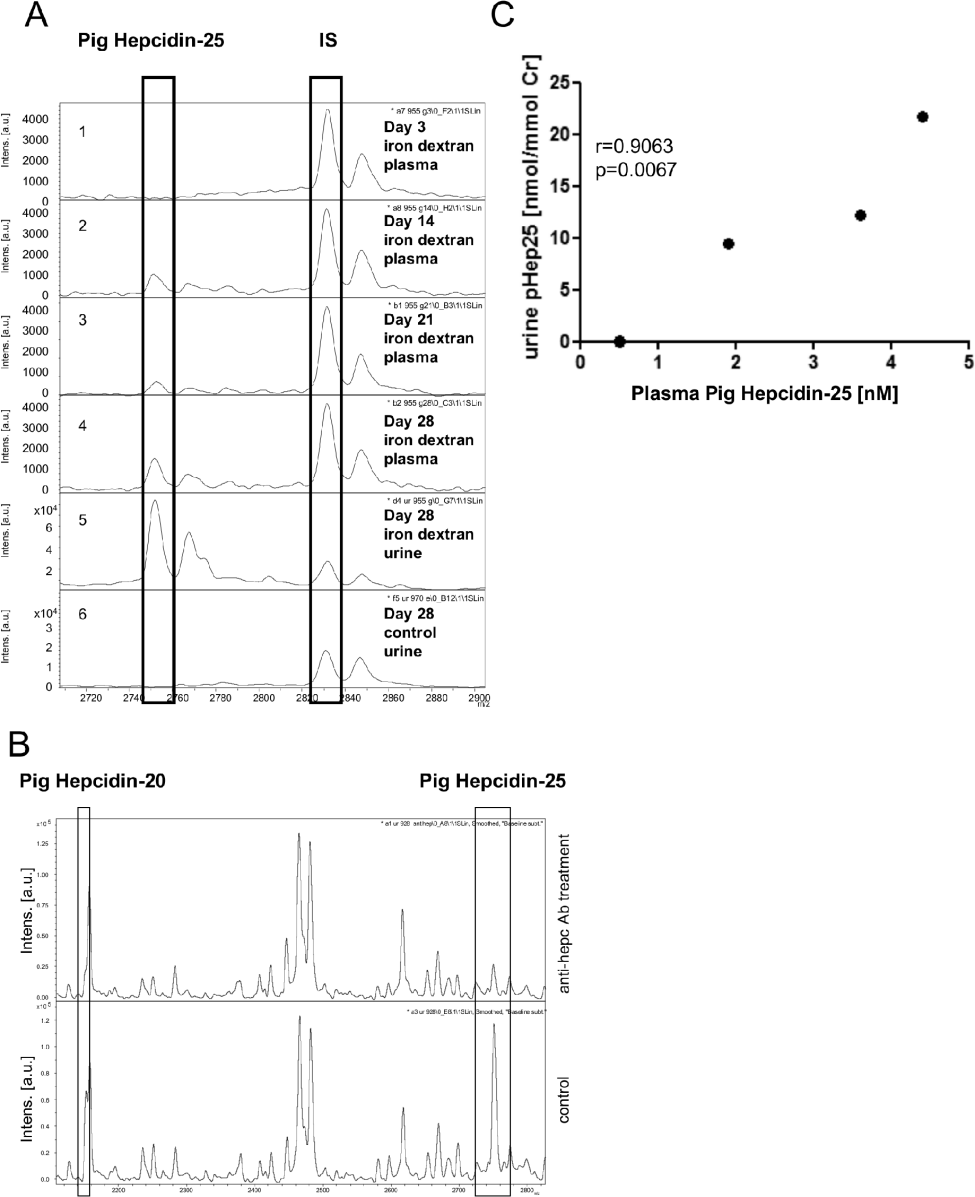
WCX-TOF MS analysis of urine porcine hepcidin. (A) Urine and plasma pig Hepcidin-25 quantification by mass spectrometry. Hepcidin-25 measurements in piglet plasma were performed by peptide enrichment through weak cation exchange chromatography coupled to time-of-flight mass spectrometry (WCX-TOF MS) [15]. The spectra (1–4) illustrate the appearance of hepcidin-25 in plasma on day 3, 14, 21 and 28 upon iron dextran injection to piglets on day 3 after birth. Spectra 5 and 6 show hepcidin-25 in the urine of piglets on day 28. Spectrum 5 corresponds to piglet injected with iron dextran on day 3 and spectrum 6 corresponds to non-supplemented, control piglet. IS—internal standard. (B) Immune capture of pig hepcidin-25 and isoform hepcidin-20 in piglet urine. Pig urine was incubated with anti-hepcidin overnight at 4°C, and subsequently hepcidin was measured by WCX-TOF MS. Hepcidin was almost not detectable in the urine sample incubated with anti-hepcidin antibody (upper spectrum); in the spectrum below (pig urine without anti-hepcidin antibody) both a high hepcidin-25 peak and a hepcidin-20 peak (mass 2152 Da) are present. (C) Correlation of pig hepcidin-25 in plasma with pig hepcidin-25 in urine measured with WCX-TOF-MS in piglets injected with iron dextran. Data from 4 measurements of urine and 4 measurements of plasma samples from day 28 were used for the statistical calculations. Spearman correlation r 0.9063, p = 0.0067.

Due to its small size (2.7 kDa), hepcidin molecule is in part eliminated from blood by glomerular filtration, but then it is taken up and degraded in the proximal tubule. A small fraction of the filtered hepcidin passes intact into the urine where it is readily detectable. To verify the usefulness of urine hepcidin as a marker of iron status in piglets, we quantified hepcidin-25 in urine samples collected from 28-day old iron-deficient and iron-replete piglets using the previously described method for pig plasma hepcidin measurement (Fig 1). Fig 1A shows the over-time increase of a mass peak of m/z 2749 (which correlates to the m/z of human hepcidin-25), in the urine profiles of iron dextran-treated pigs, but not in that of control animals. Using immunocapture with an anti-hepcidin antibody, we were able to remove this particular mass peak from the urine profile, confirming its identity as pig hepcidin-25 (Fig 1B). The concentration of urinary pig hepcidin-25, corrected for urine creatinine concentration, reflected plasma pig hepcidin-25 concentrations as determined by Spearman rank correlation (Fig 1C).

**Fig 2:**
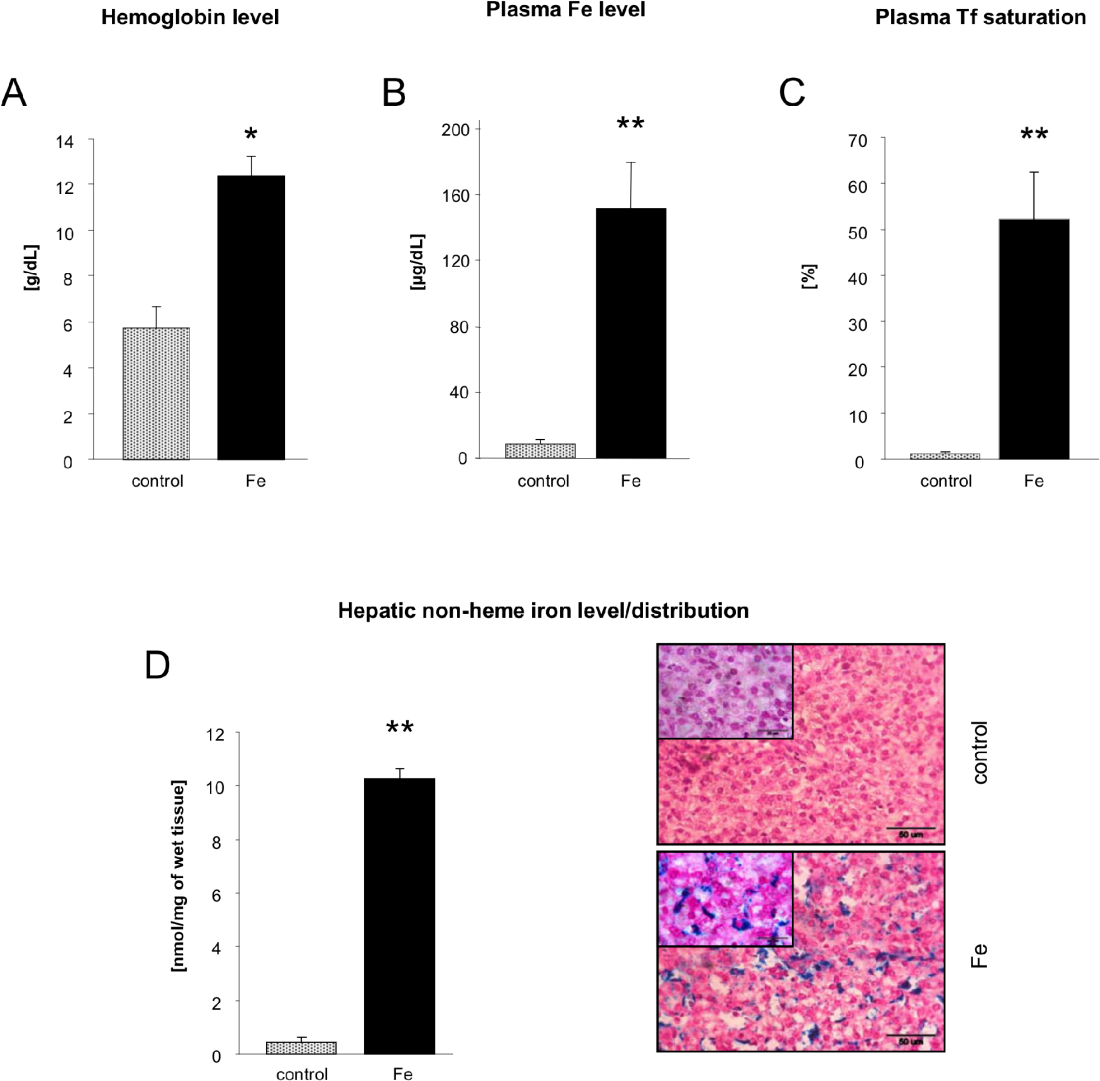
Hemoglobin levels, transferrin saturation, plasma iron level and hepatic non-heme iron content/distribution in control and iron supplemented piglets. (A) Hemoglobin level. Values are expressed as the mean ± S.D. for liver samples obtained from 7 piglets from each group. (B) Plasma iron levels and (C) iron transferrin saturation were measured and calculated as described in Material and Methods. Values are expressed as the mean ± S.D. for liver samples obtained from 7 piglets from each group. (D) Hepatic non-heme iron content and distribution were measured and examined as described in Materials and Methods. A high magnifications present deposits of iron or its absence in iron dextran injected and control piglets, respectively. Values are expressed as the mean ± S.D. for liver samples obtained from 7 piglets from each group. Statistically significant differences are indicated (*P≤0.05; **P≤0.01).

Iron was the first biological factor shown to induce hepcidin expression [2] and there is an experimental evidence that both high saturation of plasma transferrin with iron (calculated as the ratio of plasma iron to total iron-binding capacity) [23] and hepatic iron loading [2] stimulate hepcidin synthesis. The rationale for choosing 28-day old iron-deficient and iron-supplemented piglets for our study was that those animals display quantitatively two opposite iron metabolism profiles as evidenced by large differences in hemoglobin concentration (5.73±0,77 *vs* 12.37±0.61 g/dL) (Fig 2A), plasma iron level (8.85±2,49 *vs* 151.63±27.85) (Fig 2B), transferrin saturation with iron (1.22±0.43 *vs* 52.18±10.41%) (Fig 2C) and hepatic non-heme iron content (0.24±0.07 *vs* 10.30±0.37 mmol/mg) (Fig 2D; left hand panel). Accordingly, microscopic analysis of liver sections stained for non-heme iron with Perls’ Prussian Blue shows heavy iron deposits in piglets injected with iron dextran whereas in anemic piglets hepatic non-heme iron was not detected (Fig 2D; right hand panel). Indeed, hematological and biochemical iron indicators demonstrate dramatic deterioration of iron status leading to a severe anemia when no supplemental iron is administered to piglets. On the other hand, our results confirm the efficacy of routinely used iron therapy with FeDex leading to the improvement of iron parameters and thus curing iron deficiency. Such opposite conditions of iron status are useful for the validation of the urine hepcidin-25 assessment method in pigs. Of importance, an advantage of the pig model for testing urine hepcidin as an iron status marker is due to the fact that iron deficiency is the only cause of anemia in piglets. In principle, other concurring causes known to increase hepcidin expression such as inflammation [24] are not involved. Accordingly, *intra vitam* and *post mortem* veterinary examination of piglets used in the study did not reveal any symptoms of inflammation. Thus, pig anemia may be considered as a homogenous model of IDA, which has nothing to do with the anemia of chronic disease, and therefore the modulation of urinary hepcidin in this condition is inflammation-independent.

**Fig 3:**
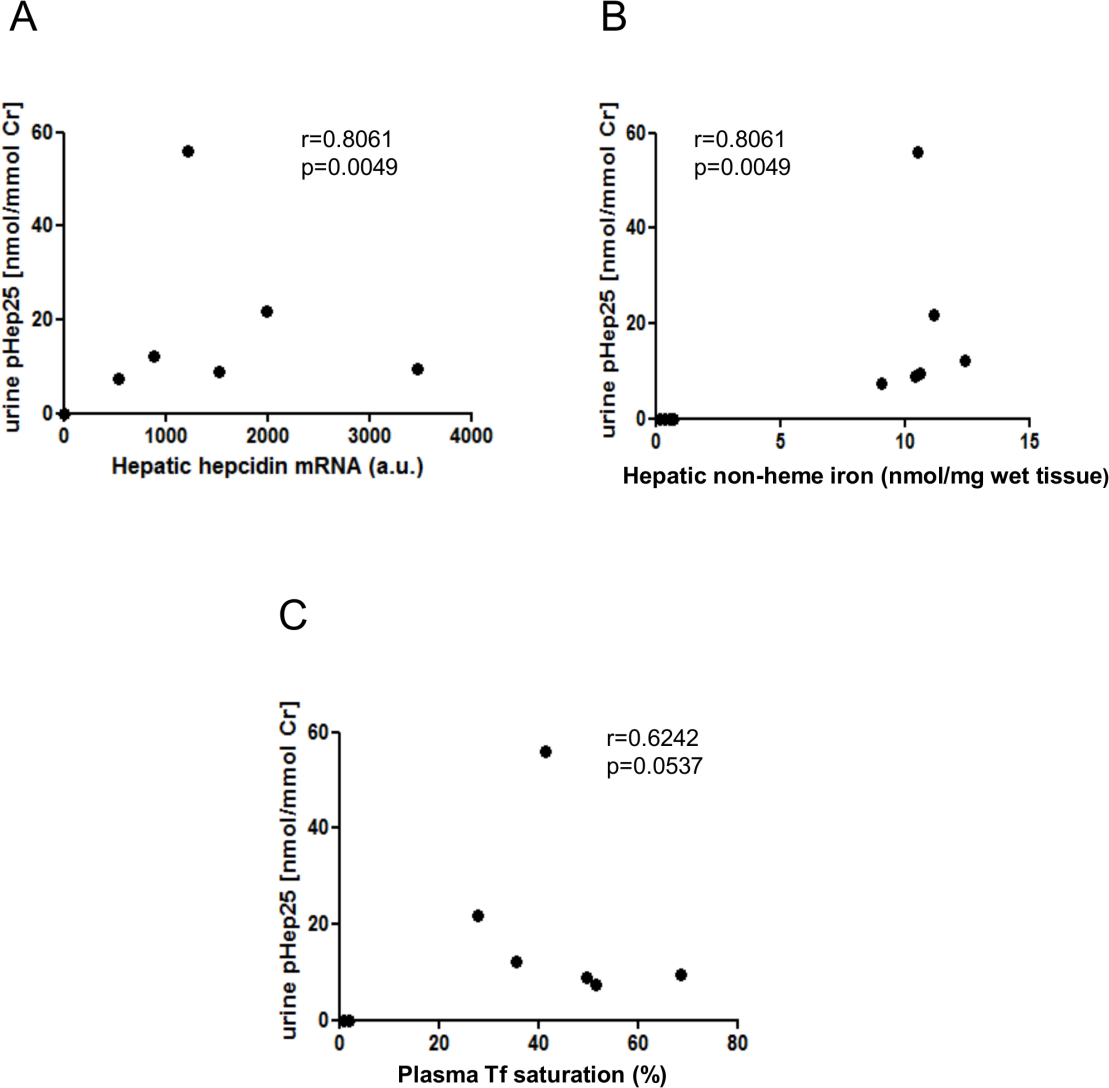
Correlations between urine hepcidin and hepatic/plasma hepcidin expression, iron transferrin saturation and hepatic non-heme iron content. Urine hepcidin-25 levels were measured in control and iron supplemented piglets, and plotted against: (A) hepcidin mRNA levels, Spearman correlation r = 0.8061, p = 0.0049; (B) hepatic non-heme iron content, Spearman correlation r = 0.8061, p = 0.0049; (C) hepatic non-heme iron content, Spearman correlation r = 0.6242, p = 0.0537. In each correlation 12 couples of examined parameters on day 28 were used for the statistical calculations.

Urine hepcidin-25 concentrations measured in anemic and iron-supplemented piglets were 0.045 and 19.25 nmol/mmol creatinine, respectively (Fig 2C). Considering the fact that piglets supplemented with FeDex accumulated toxic amount of iron in the liver as we demonstrated previously [14], this difference between piglets from two experimental groups is much greater than that found in anemic and iron-deficient patients [25]. Importantly, urine hepcidin-25 levels in both groups of piglets were highly correlated with hepatic non-heme iron content (r = 0.8061; Fig 3B) and to a lesser degree with iron transferrin saturation (r = 0.6242; Fig 3C).

**Fig 4:**
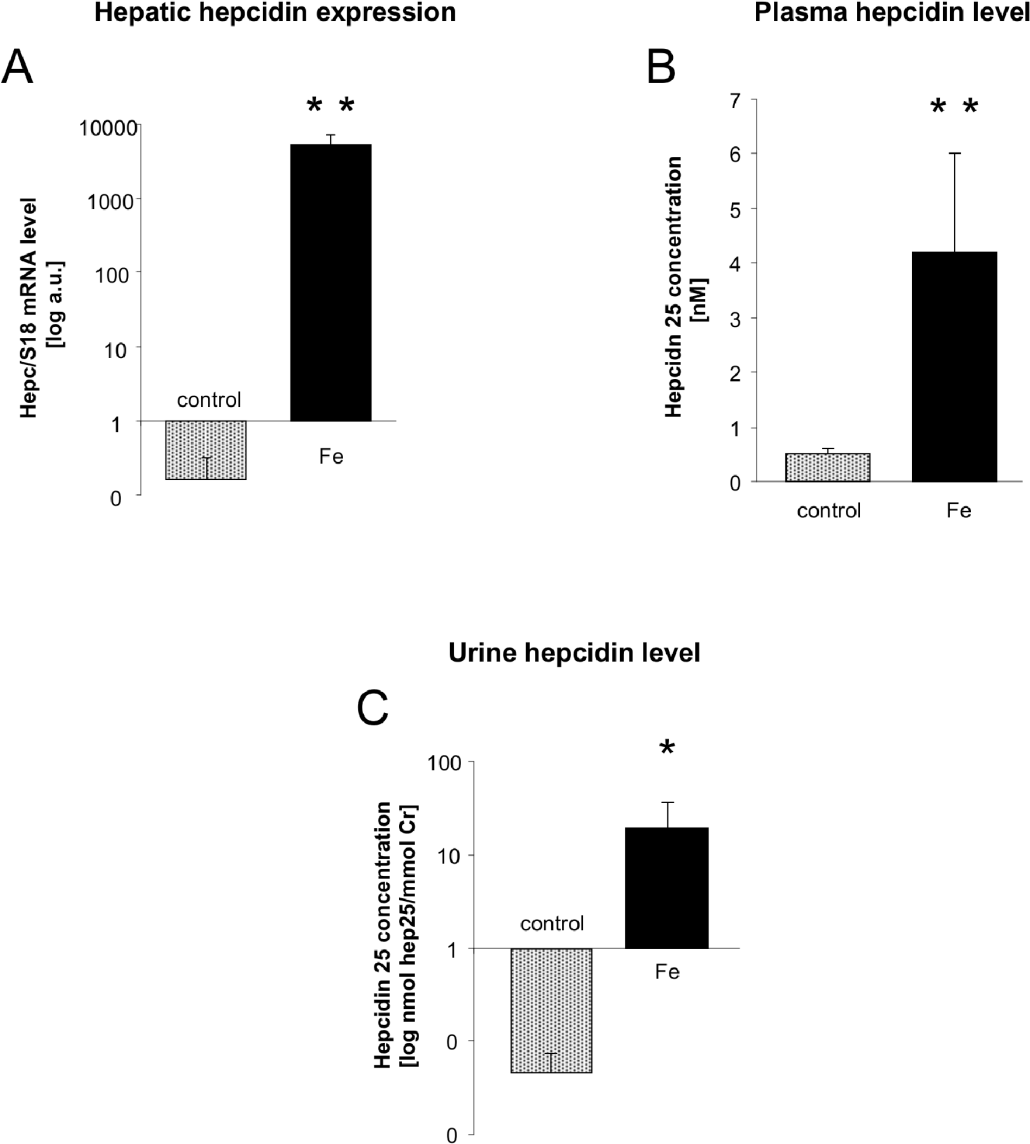
Hepatic hepcidin mRNA expression and plasma and urine hepcidin concentrations in control and iron supplemented piglets. (A) Real-time quantitative PCR analysis of hepcidin mRNA expression in the liver of control and iron-supplemented piglets. The histogram displays hepcidin mRNA levels in arbitrary units (mean ± S.D., n = 7). (B) Plasma hepcidin levels determined by the method described recently [15]. (C) Urine hepcidin levels determined by the WCX-TOF MS method described under materials and methods section. Values shown in (B) and (C) represent the mean of plasma and urine hepcidin levels of 6 and 6 samples from control and iron supplemented piglets, respectively. Statistically significant differences are indicated (* P≤0.05; ** P≤0.01).

Advantages and limitations of using urine hepcidin as marker of iron status have been exhaustively reviewed by [7]. An important criterion of its reliability is the correlation between urine hepcidin concentration and plasma level of biologically active 25-amino acid peptide. Urine hepcidin quantification in piglets may be helpful for understanding hepcidin fate, starting from the transcriptional induction of *Hamp* gene in the liver, through hepcidin secretion into the bloodstream and finally its filtration by renal glomeruli to urine. In our study, we provide a comprehensive evaluation of hepcidin expression in piglets including its mRNA abundance in the liver (Fig 4A), plasma (Fig 4B) and urine hepcidin-25 concentrations (Fig 4C). We found that urine hepcidin-25 was highly correlated with hepatic hepcidin mRNA abundance (r = 0.8061; Fig 3A). Accordingly, a significant positive correlation between urine and serum hepcidin was reported in healthy controls and patients with perturbations of iron metabolism [25], [26], [27] as well as in transgenic mouse models of iron overload and iron deficiency [28]. However, urine hepcidin concentrations may not always accurately reflect serum hepcidin concentrations [29]. We cannot exclude that the hepcidin derived from kidney (produced by tubular epithelial cells [30], [31] and/or removed from the urine by tubular reabsorption [30], [31] contributes to the amount of hepcidin-25 measured in urine samples collected from iron-supplemented piglets. Immunohistochemical analysis performed on mouse, rat and human kidneys showed that within the nephron the bioactive form of hepcidin is strongly expressed in the cortical thick ascending limb (cTAL) and connecting tubules. Moreover, in the epithelial cells of the cTAL, positive immunoreactive signal was localized in the apical part of the cells, suggesting that hepcidin is released to the urine. Release of hepcidin synthesized in the kidney into the urine was confirmed by immunoblot and ELISA analyses [30]. Several studies indicate positive correlation between iron status in the kidney and renal hepcidin expression [32], [31], [33], [34]. In particular, recent study on genetic mouse models of iron overload (hepcidin and hemojuvelin knockout mice) has indicated that there is a positive correlation between the extent of iron accumulation in the renal tubules and the degree of *Hamp* gene expression [34]. In the light of these data, it is conceivable that striking differences in urine hepcidin levels observed between anemic and iron-supplemented piglets are also due to the opposite patterns of renal iron status. However, although the renal iron content was 2-fold higher in iron supplemented piglets compared to controls, the abundance of hepcidin mRNA in the kidney did not vary between animals from both groups (data not shown). Thus, we can assume that the influence of hepcidin synthesized in the kidney on the concentration of this peptide measured in urine was negligible.

In conclusion, this study supports the use of WCX-TOF MS for measuring urine hepcidin levels as means to assess iron status in piglets. On the basis of our results young pigs emerge as an appropriate animal model, in which measurement of hepcidin in urine accurately reflects changes in iron status occurring specifically in response to iron therapy, in the absence of overlapping effects of inflammation. Considering the fact that the pig is a major biomedical mammalian model for human studies, investigating piglet iron deficiency anemia may be helpful in understanding the molecular pathophysiological mechanisms of this disorder in pre-term human neonates displaying very low iron content in their liver.
